# Effects of cocoa extract supplementation on physical performance measures: results from the randomized controlled COcoa Supplement and Multivitamin Outcomes study

**DOI:** 10.1093/jbmrpl/ziag041

**Published:** 2026-03-18

**Authors:** Sharon H Chou, Nancy Cook, Eunjung Kim, Gregory Kotler, David A Ganz, Peggy M Cawthon, Allison Clar, Aladdin H Shadyab, JoAnn E Manson, Howard D Sesso, Carolyn J Crandall, Meryl LeBoff

**Affiliations:** Division of Endocrinology, Diabetes and Hypertension, Brigham and Women’s Hospital, Boston, MA 02115, United States; Harvard Medical School, Boston, MA 02115, United States; Harvard Medical School, Boston, MA 02115, United States; Division of Preventive Medicine, Brigham and Women’s Hospital, Boston, MA 02115, United States; Department of Epidemiology, Harvard T.H. Chan School of Public Health, Boston, MA 02115, United States; Harvard Medical School, Boston, MA 02115, United States; Division of Preventive Medicine, Brigham and Women’s Hospital, Boston, MA 02115, United States; Department of Epidemiology, Harvard T.H. Chan School of Public Health, Boston, MA 02115, United States; Harvard Medical School, Boston, MA 02115, United States; Division of Preventive Medicine, Brigham and Women’s Hospital, Boston, MA 02115, United States; Department of Epidemiology, Harvard T.H. Chan School of Public Health, Boston, MA 02115, United States; Department of Medicine, David Geffen School of Medicine at University of California, Los Angeles, CA 90095, United States; Geriatric Research, Education and Clinical Center, Veterans Affairs Greater Los Angeles, CA 90073, United States; RAND, Santa Monica, CA 90401, United States; Department of Epidemiology and Biostatistics, University of California San Francisco, San Francisco, CA 94115, United States; California Pacific Medical Center, Research Institute, San Francisco, CA 94143, United States; Harvard Medical School, Boston, MA 02115, United States; Division of Preventive Medicine, Brigham and Women’s Hospital, Boston, MA 02115, United States; Department of Epidemiology, Harvard T.H. Chan School of Public Health, Boston, MA 02115, United States; Herbert Wertheim School of Public Health and Human Longevity Science, University of California San Diego, La Jolla, CA 92093, United States; Division of Geriatrics, Gerontology, and Palliative Care, Department of Medicine, University of California San Diego, La Jolla, CA 92093, United States; Harvard Medical School, Boston, MA 02115, United States; Division of Preventive Medicine, Brigham and Women’s Hospital, Boston, MA 02115, United States; Department of Epidemiology, Harvard T.H. Chan School of Public Health, Boston, MA 02115, United States; Harvard Medical School, Boston, MA 02115, United States; Division of Preventive Medicine, Brigham and Women’s Hospital, Boston, MA 02115, United States; Department of Epidemiology, Harvard T.H. Chan School of Public Health, Boston, MA 02115, United States; Division of General Internal Medicine and Health Services Research, Department of Medicine, David Geffen School of Medicine at University of California, Los Angeles, CA 90024, United States; Division of Endocrinology, Diabetes and Hypertension, Brigham and Women’s Hospital, Boston, MA 02115, United States; Harvard Medical School, Boston, MA 02115, United States

**Keywords:** cocoa extract, flavanol, multivitamin/multimineral, physical performance, grip strength, walking speed, Short Physical Performance Battery

## Abstract

Poor physical performance is associated with a higher risk of falls, fractures, and premature death among older adults. We determined whether supplementation with cocoa extract vs placebo or multivitamin/multimineral (MVM) vs placebo for 2 yr benefited physical performance measures. The COcoa Supplement and Multivitamin Outcomes Study was a double-blinded, placebo-controlled randomized trial of supplemental cocoa extract and/or MVM vs placebo for the primary prevention of cardiovascular disease and cancer in 21 442 US adults. This ancillary study was completed in a New England sub-cohort that underwent physical performance measurements, including grip strength, walking speed, standing balance, repeated chair stands, and timed-up and go (TUG) test, at baseline and 2-yr follow-up. In the clinic sub-cohort (*n* = 603) with a mean (±SD) age of 69.7 ± 5.5 yr and 49.3% women, supplemental cocoa extract, compared to cocoa-extract placebo, did not affect 2-yr changes in our primary pre-specified, co-equal outcomes of grip strength, walking speed, and the Short Physical Performance Battery (composite of walking speed, standing balance, and chair stands) or in secondary outcomes of standing balance, chair stands, or TUG tests. No effect modification by baseline characteristics of age, sex, body mass index, or randomization to MVM supplementation was observed. In parallel analyses, MVM, compared to MVM placebo, also did not affect physical performance measures. Supplementation with cocoa extract did not prevent age-related declines in physical performance measures in older ambulatory US men and women.

## Introduction

In the United States, there are more than 3 million emergency department visits per year for falls in adults 65 yr and older, and falls are the leading cause of death due to injury in this age group.^1^ In total, medical costs for falls exceed $80 billion/yr in the United States. Physical performance measures predict fall risk.^2^ Weak grip strength, slow walking speed, and other measures of poor physical performance have been associated with recurrent falls, hospitalization, and fractures.^3–8^ Given the personal and economic burden, safe and effective interventions are needed to prevent falls and maintain good physical function in older adults.

Preclinical data suggest that dietary flavanols or (−)-epicatechins, which are enriched in cocoa, may have benefits on muscle function by improving blood flow, decreasing oxidative stress, and stimulating mitochondrial biogenesis.^9–11^ In small clinical studies of short duration, ranging from 6 wk to 6 mo, supplements with (−)-epicatechin or cocoa flavanol products have been found to improve grip strength and walking speed.^11–18^ However, there are no large, long-term randomized controlled trials (RCTs) investigating the effects of a standardized cocoa flavanol formulation on physical performance measures.

Although multivitamin/multimineral (MVM) use is high in athletes and US military personnel,^19^^,^^20^ the sparse studies on the effects of MVM on physical performance have shown no benefit in active adults.^21^^,^^22^ One small trial conducted in older adults (≥70 yr old who were institutionalized) found that a multivitamin beverage did not improve physical performance.^23^ Since MVMs are widely used among older adults,^24^ a better understanding of the impact of these supplements on age-related declines in physical function is needed.

The COcoa Supplement and Multivitamin Outcomes Study (COSMOS) was a randomized, double-blind, placebo-controlled trial that investigated the effects of a cocoa extract supplement and/or an MVM on the primary prevention of cardiovascular disease and invasive cancer in 21 442 US participants. COSMOS featured a hybrid design and also included a clinic sub-cohort of 603 participants from the New England region for detailed, in-person phenotyping for mechanistic studies.^25^ The ancillary study *COSMOS: Effects on Falls and Physical Performance* (NCT05232669) was designed to investigate the effects of cocoa extract supplementation, compared to placebo, on falls (primary aim) and self-reported fractures (tertiary aim) in the overall cohort and on physical performance (secondary aim) in the clinic sub-cohort.^26^ We have previously reported that supplemental cocoa extract did not reduce the incidence of self-reported fractures over a median duration of 3.6 yr in the overall COSMOS cohort of 21 442 older adults.^27^ We herein report the effects of a cocoa extract supplement, compared to placebo, on 2-yr changes in physical performance measures in the clinic sub-cohort of 603 older ambulatory adults. We also examined the effects of an MVM supplement on physical performance.

## Materials and methods

The parent COSMOS trial randomized participants across the United States to a cocoa extract supplement (2 capsules/d containing a total of 500 mg/d of flavanols, including 80 mg (−)-epicatechin, ~50 mg theobromine, and ~15 mg caffeine [Mars Edge]) and/or an MVM (Centrum Silver; Pfizer Consumer Healthcare, now Haleon) in a 2 × 2 factorial design with respective placebos, using a computer-generated permuted block approach, which has been previously detailed.^25^^,^^28^^,^^29^ Eligible participants were women aged ≥65 yr and men aged ≥60 yr who had no history of myocardial infarction or stroke in their lifetime and no history of cancer (except non-melanoma skin cancer) in the past 2 yr. Participants who had self-reported taking ≥3/4 of study pills during a 2-mo placebo run-in phase were eligible for randomization. A total of 21 442 participants were randomized into COSMOS from April 2016 to March 2018 and treated for a median of 3.6 yr. For the duration of the trial, participants were asked not to take their own cocoa extract or MVM supplements. Participants self-reported health history, medication and supplement use, dietary patterns, and physical activity levels in questionnaires.

This study was performed in a clinical subset of COSMOS participants. Participants were eligible for the clinic sub-cohort if they participated in the parent trial, were generally healthy, and were ambulatory. Eligible participants who lived ≤50 miles of Boston were invited for detailed in-person visits, including physical performance measures, at the Center for Clinical Investigations under the Harvard Catalyst Clinical and Translational Science Center in Boston, MA. A total of 603 participants completed baseline visits, and 535 participants completed 2-yr follow up visits (88.7% retention), which concluded in November 2020. Physical performance measures, using standardized protocols, included gait speed, grip strength, timed up and go (TUG) test, chair stands, and standing balance tests. Gait speed was assessed over 6 m at normal and fast paces, using a rolling start. The walking course was 7 m in length, and participants were timed as they walked between the 0.5 and 6.5 m marks. Grip strength testing of the dominant hand was assessed with a JAMAR Plus+ Digital Hand Dynamometer (Sammons Preston Rolyan, Bolingbrook, IL, USA).^30^^,^^31^ For the TUG test, participants were timed as they stood up from a chair, walked 3 m, turned around, and returned to sit in the chair. For the standing balance tests, participants were asked to hold 3 basic standing positions for 10 s each: feet side-by-side, semi-tandem (toe by mid-foot), and full tandem stand (heel-to-toe), and the total time for all 3 positions was used for analyses. For chair stands, participants were timed as they stood and sat down, with arms folded, on a straight-back chair 5 times. Gait speed, standing balance and chair stands are components of the Short Physical Performance Battery (SPPB), scored from 0 to 12, with 12 indicating the best physical performance. Since gait speed in COSMOS was calculated over 6 m, the SPPB point system for the 3-m walk was converted to speed (ie, 1 point for <0.46 m/s, 2 points for 0.46-0.64 m/s, 3 points for 0.65-0.83 m/s, and 4 points for >0.83 m/s). All performance measures were assessed twice at each visit with the mean score used for statistical analysis, except for balance tests, which were assessed once at each visit.

To evaluate compliance with the cocoa extract intervention, participants also provided urine specimens at baseline and 2-yr follow-up for measurement of 5-(3, 4-dihydroxyphenyl)-γ-valerolactone-3 /4-sulphate (gVL3S) and gVL-3 /4-O-glucuronide metabolites (gVLM), biomarkers of flavanol intake, which was analyzed by ultra-performance LC-tandem mass spectrometry.^32^

This ancillary study was approved by the Institutional Review Board of Brigham and Women’s Hospital, and all participants provided written informed consent.

In this ancillary study *COSMOS: Effects on Falls and Physical Performance*, the effect of supplemental cocoa extract, compared to placebo, on 2-yr changes in physical performance in the clinic sub-cohort was the secondary aim. Our pre-specified primary outcomes for this aim were changes in grip strength, walking speed and SPPB score. Grip strength adjusted for BMI, which is recognized by the Sarcopenia Definition and Outcomes Consortium, was also pre-specified as a secondary sensitivity analysis.^33^ Secondary outcomes included changes in normal walking speed, fast walking speed, standing balance, chair stands, and TUG test. We examined treatment effects in an intention-to-treat fashion. Participants randomized to cocoa extract with and without MVM supplementation were compared to participants randomized to cocoa extract-placebo with and without MVM supplementation. Although the sample size of the clinic sub-cohort was determined by the parent COSMOS trial, we expected to have 80% power to detect differences of 1.03 kg for grip strength, 0.03 m/s for walking speed, and a score of 0.34 for SPPB, assuming an alpha level of 0.05. The estimates of variation over time used in these power calculations were based on changes in physical performance measures observed in the placebo group in the VITamin D and OmegA-3 TriaL, assuming 603 participants at baseline and 10% loss to follow-up.^34^ Calculation of post hoc power revealed 80% power to detect differences of 1.32 kg for grip strength, 0.03 m/s for walking speed, and a score of 0.25 for SPPB.

Baseline characteristics according to randomized treatment assignment were first compared to verify balance across intervention groups among the clinic sub-cohort. Chi-square tests were used to compare proportions, and tests for trend across ordinal variables were used. T-tests and analysis of variance (or the Wilcoxon rank sum and Kruskal Wallis tests if non-normal) were used to compare continuous variables across intervention groups.

The effect of cocoa extract supplementation on 2-yr changes in measures of physical performance was adjusted for age, sex, and randomization to active MVM supplementation. Missing data were assumed to be missing at random. All available participant data were analyzed to help prevent bias due to missing data. Repeated measures analysis with an unstructured variance matrix was used with all physical performance measures as outcomes. A time by treatment interaction was used to test intervention effects. Finally, we explored subgroup effects and effect modification by age, sex, BMI, leisure-time physical activity, history of fall(s) in the year prior to randomization, self-reported general health, randomization to MVM, and baseline urinary gVLM using 3-way time × treatment × subgroup interactions. Parallel analyses were performed for MVM vs placebo.

Statistical analyses were performed using SAS 9.4 (SAS Institute, Cary, NC). *p* values < .05 were considered statistically significant and not adjusted for multiple hypotheses testing. Thus, secondary and subgroup analyses should be interpreted with caution.

## Results

603 participants completed physical performance measures at baseline, and 535 participants returned for 2-yr follow-up (88.7% retention)(Figure S1). Baseline characteristics of the clinic sub-cohort are shown in Table 1. Mean (±SD) age was 69.7 ± 5.5 yr and 49.3% were women. At baseline, few participants had muscle weakness or low muscle performance, as defined by the European Working Group on Sarcopenia in Older People 2 (EWGSOP2).^35^ Overall, 6.5% of men and 8.1% of women had low grip strength (<27 kg in men, <16 kg in women), 2.1% had a low SPPB score ≤ 8, and 1 participant had a TUG time longer than 20 s. No participants took longer than 15 s to complete the repeated chair stands or had gait speeds slower than ≤0.8 m/s.

**Table 1:** Baseline characteristics of the clinic sub-cohort, according to randomized cocoa extract assignment.

**Variable**	**All**	**Cocoa extract**	**Cocoa extract placebo**
**Sex, no. (%)**			
**Men**	306 (50.7%)	158 (52.8%)	148 (48.7%)
**Women**	297 (49.3%)	141 (47.2%)	156 (51.3%)
**Age, years, mean (SD)**	69.7 (5.5)	70.0 (5.7)	69.5 (5.2)
**Race, no. (%)**			
**Non-Hispanic White**	582 (96.5%)	291 (97.3%)	291 (95.7)
**African American/Black**	6 (1.0%)	3 (1.0%)	3 (1.0%)
**Hispanic**	5 (0.8%)	3 (1.0%)	2 (0.7%)
**Asian/Pacific Islander**	3 (0.5%)	2 (0.7%)	1 (0.3%)
**Multiracial/other/unknown**	7 (1.2%)	0 (0.0%)	7 (2.3%)
**BMI, kg/m** ^**2**^**, mean (SD)**	28.0 (5.2)	28.3 (5.4)	27.7 (5.0)
**BMI group, kg/m** ^**2**^**, no. (%)**			
**<18.5**	5 (0.8%)	0 (0.0%)	5 (1.6%)
**18.5-24.9**	178 (29.5%)	86 (28.8%)	92 (30.3%)
**25-29.9**	240 (39.8%)	120 (40.1%)	120 (39.5%)
**30-34.9**	116 (19.2%)	54 (18.1%)	62 (20.4%)
**35+**	64 (10.6%)	39 (13.0%)	25 (8.2%)
**History of ≥1 fall in the last year, no. (%)**	177 (29.5%)	90 (30.4%)	87 (28.6%)
**History of fragility fracture, no. (%)**	116 (19.2%)	55 (18.4%)	61 (20.1%)
**Diabetes, no. (%)**	66 (11.0%)	42 (14.0%)	24 (7.9%)
**Leisure-time physical activity and stair climbing, total MET-hours/wk, median [IQR]**	20.0 [8.1–36.6]	19.2 [7.6–36.5]	21.2 [9.3–36.6]
**Smoking, no. (%)**			
**Never**	323 (54.1%)	165 (55.9%)	158 (52.3%)
**Past**	260 (43.6%)	126 (42.7%)	134 (44.4%)
**Current**	14 (2.3%)	4 (1.4%)	10 (3.3%)
**Alcohol use, no. (%)**			
**Rarely**	131 (22.9%)	76 (27.1%)	55 (18.9%)
**Monthly**	33 (5.8%)	20 (7.1%)	13 (4.5%)
**Weekly**	226 (39.6%)	106 (37.9%)	120 (41.2%)
**Daily**	181 (31.7%)	78 (27.9%)	103 (35.4%)
**General health, no. (%)**			
**Excellent**	211 (35.6%)	98 (33.4%)	113 (37.7%)
**Very good**	294 (49.6%)	143 (48.8%)	151 (50.3%)
**Good**	79 (13.3%)	49 (16.7%)	30 (10.0%)
**Fair**	9 (1.5%)	3 (1.0%)	6 (2.0%)
**Baseline use of supplemental cocoa extract, no. (%)**	2 (0.3%)	1 (0.3%)	1 (0.3%)
**Baseline use of MVM, no. (%)**	231 (38.4%)	127 (42.5%)	104 (34.3%)
**Baseline use of supplemental vitamin D, no. (%)**	249 (41.7%)	130 (43.6%)	119 (39.8%)
**Dark chocolate intake, servings/wk, median [IQR]**	0.5 [0.0-1.0]	0.5 [0.0-1.0]	0.5 [0.0-1.0]
**Milk chocolate intake, servings/wk, median [IQR]**	0.5 [0.0-0.5]	0.5 [0.0-1.0]	0.5 [0.0-0.5]
**Baseline urinary gVLM, μmol/L, median [IQR]**	2.81 [0.67-9.45]	2.12 [0.59-9.37]	2.91 [0.77-9.72]
**Randomized to MVM, no. (%)**	289 (47.9%)	154 (51.5%)	135 (44.4%)

Abbreviations: MET, metabolic equivalent of task; IQR, interquartile range.

Baseline characteristics were well-balanced between the cocoa extract group and the cocoa-extract placebo group in terms of sex, age, race, BMI, leisure-time physical activity, tobacco use, and self-reported general health (Table 1). More participants randomized to cocoa extract had history of diabetes compared to placebo (14% vs 7.9%, respectively). Alcohol intake was more frequent in the placebo group. While there was greater use of MVM prior to the trial in the cocoa extract group, there were only 2 participants in the entire clinic sub-cohort who took cocoa extract supplements prior to the trial and there was no difference in chocolate intake or urinary gVLM, a biomarker of flavanols, at baseline between the groups.

There were no significant differences in baseline characteristics between the MVM group and the MVM placebo group (Table S1).

Over 2 yr, urinary gVLM increased from mean (±SE) of 9.5 ± 1.0 to 21.1 ± 1.8 μmol/L in the cocoa extract group. As expected, there were no changes in urinary gVLM in the cocoa-extract placebo group (8.7 ± 1.0 to 8.0 ± 1.7 μmol/L, *p* = .65, treatment effect *p* < .001).

After 2 yr, grip strength was notably weaker in all participants, similarly between the active and placebo cocoa extract groups, with change of −12 kg, *p* < .001, and − 12 kg, *p* < .001, respectively in men, and change of −6.7 kg, *p* < .001, and − 6.2 kg, *p* < .001, respectively in women (Table 2). Over 95% of the participants used the same hand (right vs left) to assess grip strength at baseline and 2-yr follow-up; only 19 participants used different hands. In exploratory analyses, a greater 2-yr decline in grip strength was observed in men than women (changes of −12 kg and −6.5 kg, respectively). Older participants (≥70 yr old) did not have greater loss in grip strength than younger participants (<70 yr old), with changes of −8.4 kg and − 10 kg, respectively.

**Table 2:** Two-year changes in physical performance measure, according to randomized cocoa extract assignment, adjusted for age, sex, and treatment group.

		**Cocoa extract**		**Cocoa extract placebo**		**Treatment effect**	
**Measure**	***N***	**Mean (SE)**	***p*-value**	**Mean (SE)**	***p*-value**	**Mean (SE)**	***p*-value**
**Grip strength, men, kg**							
**Baseline**	306	37.71 (0.63)		37.84 (0.66)			
**Year 2**	272	25.70 (0.60)		26.06 (0.62)			
**2-yr change**		−12.01 (0.47)	<.001	−11.77 (0.49)	<.001	−0.23 (0.68)	.74
**Grip strength, women, kg**							
**Baseline**	297	21.99 (0.38)		22.00 (0.36)			
**Year 2**	263	15.27 (0.35)		15.76 (0.33)			
**2-yr change**		−6.72 (0.33)	<.001	−6.24 (0.31)	<.001	−0.48 (0.45)	.29
**SPPB score**							
**Baseline**	569	11.05 (0.06)		11.19 (0.06)			
**Year 2**	509	10.96 (0.07)		10.97 (0.07)			
**2-yr change**		−0.09 (0.07)	.19	−0.22 (0.06)	<.001	0.13 (0.09)	.15
**Normal walking speed, m/s**							
**Baseline**	599	1.19 (0.01)		1.20 (0.01)			
**Year 2**	529	1.16 (0.01)		1.16 (0.01)			
**2-yr change**		−0.03 (0.01)	<.001	−0.04 (0.01)	<.001	0.01 (0.01)	.35
**Fast walking speed, m/s**							
**Baseline**	587	1.66 (0.01)		1.68 (0.01)			
**Year 2**	522	1.64 (0.02)		1.65 (0.01)			
**2-yr change**		−0.02 (0.01)	.045	−0.03 (0.01)	.01	0.01 (0.02)	.72
**Standing balance, s**							
**Baseline**	595	28.98 (0.16)		29.08 (0.16)			
**Year 2**	526	29.43 (0.14)		29.33 (0.13)			
**2-yr change**		0.46 (0.17)	.007	0.25 (0.16)	.13	0.20 (0.24)	.39
**Chair stands, s**							
**Baseline**	573	11.48 (0.15)		11.33 (0.14)			
**Year 2**	513	12.03 (0.20)		11.89 (0.20)			
**2-yr change**		0.55 (0.18)	.003	0.56 (0.18)	.002	−0.01 (0.25)	.98
**Timed up and go, s**							
**Baseline**	597	8.36 (0.08)		8.31 (0.08)			
**Year 2**	522	7.95 (0.09)		7.86 (0.09)			
**2-yr change**		−0.41 (0.08)	<.001	−0.45 (0.08)	<.001	0.04 (0.11)	.75

Abbreviation: SPPB, Short Physical Performance Battery.

There were minimal declines in walking speeds (≤−0.04 m/s) and increased time for the completion of chair stands (<0.8 s) across all treatment groups. Participants were slightly faster in completing the TUG tests, though the change was clinically very small (<−0.5 s).

Supplementation with cocoa extract for 2 yr, compared to the cocoa-extract placebo, did not affect physical performance measures in our clinic sub-cohort (Table 2). We found no benefit of supplemental cocoa extract on our primary pre-specified outcomes of grip strength, walking speed, and SPPB or secondary outcomes of standing balance, chair stands, or TUG tests. As a pre-specified sensitivity analysis, we also examined changes in grip strength adjusted for BMI, as acknowledged by the Sarcopenia Definition and Outcomes Consortium,^33^ and also did not find any differences in 2-yr changes between the cocoa extract and cocoa-extract placebo groups (Table S3).

In subgroup analyses, there was no effect modification for the effect of cocoa extract supplementation on grip strength, normal gait speed, or SPPB, by sex, age (divided at the median), BMI (divided at the median), baseline urinary gVLM (divided at the median), leisure-time physical activity by metabolic equivalent of task (divided at the median), self-reported general health, or randomization to MVM (Table 3). Among participants who had a fall within the year before the start of the study, those who were randomized to cocoa extract had less of a decline in grip strength than those randomized to cocoa-extract placebo, while participants who did not have a fall had a greater decline in grip strength with cocoa extract than with cocoa-extract placebo (*p* for interaction <.001). Among the participants who had a prior fall at baseline, 63% were women and 37% were men. In exploratory analyses, men with a prior fall were found to have a greater decline in grip strength with placebo than with cocoa extract supplementation (−13.53 [0.85] kg and −10.28 [1.00] kg, respectively; *p*-value, treatment effect, .016), while women with a prior fall had similar declines in grip strength in the 2 treatment arms (−6.34 [0.56] kg and −6.48 [0.51] kg, respectively; *p*-value, treatment effect, .85; *p* for interaction .016). Since a single fall may be a spurious event and recurrent falls may suggest ongoing functional limitation, subgroup analyses were also performed for participants with 2 or more falls in the prior year at baseline. Participants who had 2 or more falls in the prior year also had a greater decline in grip strength with placebo than cocoa extract, while participants without 2 or more falls had a slightly greater decline in grip strength with cocoa extract than with placebo (*p* for interaction .01).

**Table 3:** Effect of cocoa extract supplementation vs placebo on physical performance measures in subgroups.

	Cocoa extract			Cocoa extract placebo			*p*-value, treatment effect	*P* for interaction
Subgroup	*N*	2-yr change (SE)	*p*-value	*N*	2-yr change (SE)	*p*-value		
**Grip strength, kg**								
**Sex**								.72
**Men**	158	−12.01 (0.47)	<.001	148	−11.77 (0.49)	<.001	0.74	
**Women**	141	−6.72 (0.33)	<.001	156	−6.24 (0.31)	<.001	0.29	
**Age**								.43
**<Median (69.2 yr)**	143	−10.77 (0.49)	<.001	158	−9.76 (0.47)	<.001	0.14	
**≥Median**	156	−8.32 (0.44)	<.001	146	−8.09 (0.44)	<.001	0.71	
**BMI**								.16
**<Median (26.5 kg/m**^**2**^**)**	143	−9.25 (0.47)	<.001	158	−8.07 (0.44)	<.001	0.06	
**≥Median**	156	−9.77 (0.47)	<.001	146	−9.91 (0.49)	<.001	0.84	
**Baseline urinary gVLM**								.58
**<Median (2.8 μmol/L)**	127	−9.15 (0.50)	<.001	112	−8.79 (0.53)	<.001	0.62	
**≥Median**	117	10.35 (0.57)	<.001	122	−9.38 (0.55)	<.001	0.22	
**Leisure-time physical activity and stair climbing, total MET**								.48
**<Median (20.0 h/wk)**	155	−8.57 (0.45)	<.001	144	−8.33 (0.46)	<.001	0.70	
**≥Median**	142	−10.45 (0.49)	<.001	158	−9.54 (0.47)	<.001	0.18	
**History of ≥1 fall in the last year**								<.001
**Yes**	90	−7.66 (0.57)	<.001	87	−9.49 (0.57)	<.001	0.02	
**No**	206	−10.34 (0.40)	<.001	217	−8.71 (0.39)	<.001	0.004	
**History of ≥2 falls in the last year**								.01
**Yes**	31	−8.68 (1.06)	<.001	41	−11.13 (0.90)	<.001	0.08	
**No**	265	−9.65 (0.35)	<.001	263	−8.61 (0.35)	<.001	0.04	
**General health, self-reported**								.24
**Excellent**	98	−9.90 (0.58)	<.001	113	−8.84 (0.53)	<.001	0.18	
**Very good**	143	−9.30 (0.48)	<.001	151	−9.50 (0.47)	<.001	0.77	
**Good**	49	−9.29 (0.83)	<.001	30	−7.03 (1.03)	<.001	0.09	
**Fair**	3	−6.60 (3.63)	.14	6	−10.16 (2.80)	.02	0.48	
**Randomized to MVM**								.76
**Yes**	154	−9.72 (0.47)	<.001	135	−9.33 (0.49)	<.001	0.60	
**No**	145	−9.33 (0.47)	<.001	169	−8.62 (0.44)	<.001	0.27	
**SPPB score**								
**Sex**								.97
**Men**	158	−0.19 (0.08)	.02	148	−0.33 (0.09)	<.001	0.23	
**Women**	141	0.04 (0.10)	.7	156	−0.11 (0.09)	.26	0.30	
**Age**								.22
**<Median (69.2 yr)**	143	−0.14 (0.09)	.10	158	−0.16 (0.08)	.05	0.86	
**≥Median**	156	−0.03 (0.10)	.77	146	−0.28 (0.10)	.005	0.08	
**BMI**								.87
**<Median (26.5 kg/m**^**2**^**)**	143	−0.14 (0.09)	.12	158	−0.26 (0.08)	.002	0.32	
**≥Median**	156	−0.03 (0.10)	.76	146	−0.18 (0.10)	.07	0.29	
**Baseline urinary gVLM**								.88
**<Median (2.8 μmol/L)**	127	0.03 (0.11)	.77	112	−0.13 (0.12)	.27	0.32	
**≥Median**	117	−0.13 (0.10)	.20	122	−0.31 (0.09)	.001	0.18	
**Leisure-time physical activity and stair climbing, total MET**								.35
**<Median (20.0 h/wk)**	155	−0.06 (0.10)	.55	144	−0.29 (0.10)	.005	0.12	
**≥Median**	142	−0.12 (0.08)	.17	158	−0.17 (0.08)	.04	0.66	
**History of ≥1 fall in the last year**								.96
**Yes**	90	−0.19 (0.12)	.13	87	−0.33 (0.12)	.008	0.41	
**No**	206	−0.04 (0.08)	.62	217	−0.17 (0.07)	.02	0.21	
**History of ≥2 falls in the last year**								.96
**Yes**	31	−0.28 (0.23)	.22	41	−0.42 (0.19)	.04	0.64	
**No**	265	−0.06 (0.07)	.38	263	−0.19 (0.07)	.005	0.18	
**General health, self-reported**								.67
**Excellent**	98	−0.21 (0.10)	.04	113	−0.25 (0.09)	.009	0.80	
**Very good**	143	−0.01 (0.10)	.93	151	−0.18 (0.09)	.05	0.20	
**Good**	49	0.10 (0.20)	.61	30	−0.23 (0.23)	.32	0.28	
**Fair**	3	***	***	6	***	***		
**Randomized to MVM**								.10
**Yes**	154	−0.05 (0.09)	.57	135	−0.34 (0.09)	<.001	0.02	
**No**	145	−0.12 (0.10)	.22	169	−0.11 (0.09)	.20	0.97	
**Normal walking speed, m/s**								
**Sex**								.62
**Men**	158	−0.01 (0.01)	.62	148	−0.02 (0.01)	.09	0.39	
**Women**	141	−0.06 (0.01)	<.001	156	−0.07 (0.01)	<.001	0.87	
**Age**								.57
**<Median (69.2 yr)**	143	−0.02 (0.01)	.06	158	−0.03 (0.01)	.02	0.79	
**≥Median**	156	−0.04 (0.01)	<.001	146	−0.06 (0.01)	<.001	0.26	
**BMI**								.22
**<Median (26.5 kg/m**^**2**^**)**	143	−0.03 (0.01)	.02	158	−0.06 (0.01)	<.001	0.15	
**≥Median**	156	−0.03 (0.01)	.002	146	−0.03 (0.01)	.008	0.82	
**Baseline urinary gVLM**								.52
**<Median (2.8 μmol/L)**	127	−0.04 (0.01)	.003	112	−0.04 (0.01)	.006	0.96	
**≥Median**	117	−0.03 (0.01)	.03	122	−0.05 (0.01)	<.001	0.40	
**Leisure-time physical activity and stair climbing, total MET**								1.00
**<Median (20.0 h/wk)**	155	−0.04 (0.01)	.004	144	−0.04 (0.01)	<.001	0.64	
**≥Median**	142	−0.03 (0.01)	.004	158	−0.04 (0.01)	<.001	0.58	
**History of ≥1 fall in the last year**								.49
**Yes**	90	−0.02 (0.02)	.21	87	−0.04 (0.02)	.009	0.34	
**No**	206	−0.04 (0.01)	<.001	217	−0.04 (0.01)	<.001	0.74	
**History of ≥2 falls in the last year**								.72
**Yes**	31	−0.04 (0.03)	.18	41	−0.03 (0.02)	.16	0.92	
**No**	265	−0.03 (0.01)	<.001	263	−0.05 (0.01)	<.001	0.35	
**General health, self-reported**								.01
**Excellent**	98	−0.01 (0.01)	.48	113	−0.07 (0.01)	<.001	0.003	
**Very good**	143	−0.05 (0.01)	<.001	151	−0.03 (0.01)	.005	0.32	
**Good**	49	−0.04 (0.02)	.09	30	−0.00 (0.03)	.97	0.28	
**Fair**	3	−0.15 (0.07)	.13	6	−0.17 (0.07)	.10	0.85	
**Randomized to MVM**								.45
**Yes**	154	−0.03 (0.01)	.03	135	−0.05 (0.01)	<.001	0.27	
**No**	145	−0.04 (0.01)	<.001	169	−0.04 (0.01)	<.001	0.88	

Abbreviations: MET, metabolic equivalent of task; MVM, multivitamin/multimineral; gVLM, 5-(3,4-dihydroxyphenyl)-γ-valerolactone metabolites*;* SPPB, Short Physical Performance Battery.

As the COSMOS clinic sub-cohort was generally healthy and included few participants with muscle weakness or low muscle performance, physical performance measures of SPPB and overall balance tests may exhibit ceiling effects in our study population. Thus, we performed sensitivity analyses on individual balance tests of semi-tandem and tandem stand, which is the most difficult position to hold. There were no between group differences with 2-yr changes in semi-tandem stand. Participants randomized to supplemental cocoa extract exhibited improvements in the tandem stand, compared to placebo (*p* = .028)(Table S3).

Supplementation with an MVM, compared to an MVM placebo, also did not affect 2-yr changes in physical performance measures (Table S2), including grip strength adjusted for BMI (Table S3). In sensitivity analyses, there were no between group differences with 2-yr changes in semi-tandem and tandem stands (Table S3). There was also no effect modification by age, sex, prior use of MVM (discontinued during 2-mo placebo run-in phase and intervention period), other baseline characteristics, or randomization to cocoa extract (Table S4).

Potential adverse effects of cocoa extract supplementation, including nausea and upset stomach, have been previously reported by the parent COSMOS trial.^30^

## Discussion

Our study in ambulatory older men and women with a mean age of 69.7 yr found that, compared to placebo, cocoa extract supplementation did not affect 2-yr changes in physical performance measures of grip strength, walking speed, or SPPB score. Multivitamin/multimineral supplementation also did not affect 2-yr changes in physical performance measures.

Our study population was overall healthy, with a low prevalence of muscle weakness or low muscle performance, as defined by EWGSOP2,^35^ at baseline. Thus, the physical performance measures assessed in our study, particularly the SPPB, may have exhibited ceiling effects. In sensitivity analyses, we found that supplemental cocoa extract, compared to placebo, did improve the ability to hold the tandem stand position.

We had explored if cocoa extraction supplementation would benefit participants with poorer physical function, using a history of fall(s) in the year prior to randomization as subgroups. In these participants with a prior-year fall, supplemental cocoa extract lessened the decline in grip strength compared to placebo. However, this may be a chance finding as we have no explanation for why participants without a history of fall(s) in the prior year would have worsening grip strength with cocoa extract supplementation. Also, the difference in grip strength decline (Δ1.81 kg) in the participants with a prior-year fall was less than the estimated annual loss of grip strength in the placebo group overall (3.12-5.89 kg, depending on sex).

Findings from other RCTs, which were smaller and of shorter duration, indicate that cocoa may benefit high risk individuals. An RCT in older adults (*n* = 61, mean ± SD age of 75.9 ± 5.7 yr) found that daily supplementation with a cocoa powder-based beverage with 25 mg of epicatechin for 12 wk improved walking speed, grip strength, and quality of life and decreased frailty, compared to a control beverage without flavonoids.^15^ Another 8-wk RCT in 62 men with sarcopenia (mean ± SD age of 68.6 ± 2.9 yr) found that supplemental epicatechin (dosed at 1 mg/kg of body weight) improved times for the TUG test slightly (Δ0.22 s) and the appendicular muscle mass index (Δ0.34 kg/m^2^) compared to placebo, but not compared to resistance training alone.^14^ A phase II clinical trial randomized people with lower extremity peripheral artery disease (*n* = 44) to a daily cocoa beverage with 75 mg of epicatechin vs a placebo beverage and found that cocoa improved 5-min walking distance by 42.6 m.^11^ Furthermore, on calf muscle biopsies, cocoa improved mitochondrial cytochrome *c* oxidase activity, capillary density, and calf muscle perfusion and reduced the accumulation of central nuclei, an indicator of myopathy.^11^ The authors also found evidence of cocoa activating nuclear factor erythroid 2-related factor 2 (Nfr2), leading to increased antioxidants heme oxygenase-1 (HO-1) and NAD(P)H dehydrogenase [quinone] (NQO1) and protection against skeletal muscle damage.^36^

These RCTs had administered supplements with similar or lower doses of epicatechin compared to the 80 mg/d regimen used in our study. Furthermore, we do not suspect that the baseline intake of cocoa in our RCT was higher than in these earlier studies, as baseline use of supplemental cocoa extract in our clinical trial was very low at 0.3%. Chocolate intake was also minimal in our study with 0.5 servings per wk of each dark and milk chocolate, which have some flavanols but at much lower concentrations. The daily cocoa extract supplement administered in COMSOS was highly concentrated with 500 mg of flavanols including 80 mg (−)-epicatechin, which is the equivalent of 90.7 g (3.2 ounces) of dark chocolate or 771.1 g (1.7 pounds) of milk chocolate. The main difference between our study and prior RCTs is our overall healthy study population. The populations of the clinical trials that found benefits in physical performance measures were selected on the basis of peripheral artery disease,^11^^,^^13^ heart failure and diabetes,^16^^,^^18^ and sarcopenia.^14^ In subgroup analyses for COSMOS, there may have been a benefit with cocoa extract for grip strength in participants with fall(s) in the last year. Overall, however, we did not find a benefit for cocoa extract vs placebo in older or less active participants in this ambulatory sub-cohort.

The few RCTs testing an MVM for physical performance have not found a benefit for daily MVM, even in those at highest risk.^21–23^ In a small study in institutionalized participants in their eighth and ninth decades of life (*n* = 55), a multivitamin beverage did not improve physical performance as assessed by spiroergometric tests.^23^

Grip strength, including when adjusted for BMI, notably declined over 2 yr in all participants, without any difference between treatment groups. While the grip strength assessed at baseline was compatible to other cohorts, the loss of grip strength after 2 yr was more than we expected.^34^^,^^37^ The equipment and protocol, including the verbal script for study staff to instruct participants, to assess grip strength did not change during the study and were comparable across all treatment groups at each time point. There may have been a calibration issue with the equipment between the baseline and 2-yr visits. Despite the larger-than-expected 2-yr reductions in grip strength, we believe our comparisons for both COSMOS interventions are valid.

These findings complement our previously reported results that neither supplementation with cocoa extract nor an MVM reduced the incidence of self-reported fractures in the overall COSMOS cohort of 21 442 older adults over a median of 3.6 yr of follow up.^27^ Since both the overall COSMOS cohort and the clinic sub-cohort were overall healthy, our results may not apply to older individuals with medical comorbidities and/or sarcopenia.

In the overall COSMOS cohort, supplemental cocoa extract and MVM, compared to respective placebos, did not reduce the composite cardiovascular outcome or all-cause mortality, though cocoa extract reduced cardiovascular death by 27%.^28^^,^^29^ Cocoa extract and MVM supplementation, compared to respective placebos, also did not reduce the incidence of invasive cancer overall, though the MVM had a protective effect on lung cancer.^28^^,^^29^ In ancillary studies, MVM supplementation was found to have benefits on cognitive function and memory.^38–40^

Strengths of this study include a large sample size, inclusion of women and men, a long study duration, and high adherence. This is the first large, randomized trial of cocoa extract vs placebo over 2 yr on changes in physical performance. Although the sample size of the clinic sub-cohort was determined by the parent COSMOS trial, we had excellent power to determine minimal changes in physical performance measures. Despite no definitive explanation for the greater than expected decline in grip strength, the effect was similar by treatment group, and we do not expect any other bias. Because COSMOS investigated the effects of cocoa extracts in primarily community-dwelling older adults and included few participants with muscle weakness or low muscle performance, the results are not generalizable to institutionalized adults or individuals with poor physical function.

Cocoa extract or MVM supplementation for 2 yr did not slow age-related declines in physical performance in generally healthy ambulatory older adults.

## Supplementary Material

Physical_performance_supplementary_clean_ziag041

## Data Availability

Data described in the manuscript, code book, and analytic code will be made available upon request pending application and approval.

## Clinical trial registration

ClinicalTrials.gov ID NCT05232669; https://clinicaltrials.gov/study/NCT05232669; first posted on February 10, 2022.
