# Supplementary material for: Effects of cocoa extract supplementation on physical performance measures: results from the randomized controlled COcoa Supplement and Multivitamin Outcomes study
Source: JBMR Plus. 2026 Mar 18;10(5):ziag041. doi: 10.1093/jbmrpl/ziag041 (PMC13089503; doi:10.1093/jbmrpl/ziag041)
Supplement: Physical_performance_supplementary_clean_ziag041 [file physical_performance_supplementary_clean_ziag041.docx]

Supplementary Figure 1. Randomization and follow-up of participants

21,442 underwent randomization into the COSMOS trial

68 did not complete 2-year follow-up visits

535 completed physical performance measures at 2-year follow-up

603 completed physical performance measures at baseline

304 assigned to cocoa extract placebo

299 assigned to cocoa extract

603 entered the clinic sub-cohort

Supplementary Table 1. Baseline characteristics of the clinic sub-cohort, according to randomized Multivitamin/Multimineral (MVM) assignment

| Variable | All | MVM | MVM Placebo |
| --- | --- | --- | --- |
| Sex, no. (%) |  |  |  |
| Men | 306 (50.7%) | 147 (50.9%) | 159 (50.6%) |
| Women | 297 (49.3%) | 142 (49.1%) | 155 (49.4%) |
| Age, years, mean (SD) | 69.7 (5.5) | 69.5 (5.2) | 70.0 (5.8) |
| Race, no. (%) |  |  |  |
| Non-Hispanic White | 582 (96.5%) | 283 (97.9%) | 299 (95.2%) |
| African American/Black | 6 (1.0%) | 2 (0.7%) | 4 (1.3%) |
| Hispanic | 5 (0.8%) | 3 (1.0%) | 2 (0.6%) |
| Asian/Pacific Islander | 3 (0.5%) | 1 (0.3%) | 2 (0.6%) |
| Multiracial/Other/ Unknown | 7 (1.2%) | 0 (0.0%) | 7 (2.2%) |
| Body Mass Index, kg/m^2^, mean (SD) | 28.0 (5.2) | 27.8 (5.1) | 28.3 (5.2) |
| Body Mass Index group, kg/m^2^, no. (%) |  |  |  |
| <18.5 | 5 (0.8%) | 2 (0.7%) | 3 (1.0%) |
| 18.5-24.9 | 178 (29.5%) | 89 (30.8%) | 89 (28.3%) |
| 25-29.9 | 240 (39.8%) | 114 (39.4%) | 126 (40.1%) |
| 30-34.9 | 116 (19.2%) | 60 (20.8%) | 56 (17.8%) |
| 35+ | 64 (10.6%) | 24 (8.3%) | 40 (12.7%) |
| History of ≥1 fall in the last year, no. (%) | 177 (29.5%) | 86 (30.0%) | 91 (29.1%) |
| History of fragility fracture, no. (%) | 116 (19.2%) | 53 (18.3%) | 63 (20.1%) |
| Diabetes, no. (%) | 66 (11.0%) | 27 (9.3%) | 39 (12.5%) |
| Leisure-time physical activity and stair climbing, total MET-hours/week, median [IQR] | 20.0 [8.1-36.6] | 21.5 [7.4-35.8] | 18.9 [9.1-37.6] |
| Smoking, no. (%) |  |  |  |
| Never | 323 (54.1%) | 153 (53.5%) | 170 (54.7%) |
| Past | 260 (43.6%) | 125 (43.7%) | 135 (43.4%) |
| Current | 14 (2.3%) | 8 (2.8%) | 6 (1.9%) |
| Alcohol use, no. (%) |  |  |  |
| Rarely | 131 (22.9%) | 56 (20.5%) | 75 (25.2%) |
| Monthly | 33 (5.8%) | 20 (7.3%) | 13 (4.4%) |
| Weekly | 226 (39.6%) | 107 (39.2%) | 119 (39.9%) |
| Daily | 181 (31.7%) | 90 (33.0%) | 91 (30.5%) |
| General Health, no. (%) |  |  |  |
| Excellent | 211 (35.6%) | 106 (37.3%) | 105 (34.0%) |
| Very Good | 294 (49.6%) | 135 (47.5%) | 159 (51.5%) |
| Good | 79 (13.3%) | 39 (13.7%) | 40 (12.9%) |
| Fair | 9 (1.5%) | 4 (1.4%) | 5 (1.6%) |
| Baseline use of supplemental cocoa extract, no. (%) | 2 (0.3%) | 0 (0.0%) | 2 (0.6%) |
| Baseline use of MVM, no. (%) | 231 (38.4%) | 114 (39.4%) | 117 (37.4%) |
| Baseline use of supplemental vitamin D, no. (%) | 249 (41.7%) | 121 (42.5%) | 128 (41.0%) |
| Dark chocolate intake, servings/week, median [IQR] | 0.5 [0.0-1.0] | 0.5 [0.0-1.0] | 0.5 [0.0-1.0] |
| Milk chocolate intake, servings/week, median [IQR] | 0.5 [0.0-0.5] | 0.5 [0.0-1.0] | 0.5 [0.0-0.5] |
| Baseline urinary gVLM, µmol/L, median [IQR] | 2.81 [0.67-9.45] | 3.06 [0.86-10.72] | 2.36 [0.54-8.85] |
| Randomized to Cocoa Extract, no. (%) | 299 (49.6%) | 154 (53.3%) | 145 (46.2%) |

Abbreviations: MET, metabolic equivalent of task; IQR, interquartile range

Supplementary Table 2. Two-year changes in physical performance measure, according to randomized Multivitamin/Multimineral (MVM) assignment, adjusted for age, sex, and treatment group

|  |  | MVM | | MVM Placebo | | Treatment Effect | |
| --- | --- | --- | --- | --- | --- | --- | --- |
| Measure | N | Mean (SE) | p-value | Mean (SE) | p-value | Mean (SE) | p-value |
| Grip strength, men, kg | | | | | | |  |
| Baseline | 306 | 37.66 (0.66) |  | 37.90 (0.63) |  |  |  |
| Year 2 | 272 | 25.44 (0.62) |  | 26.32 (0.59) |  |  |  |
| 2-year change |  | -12.23 (0.49) | <0.001 | -11.58 (0.47) | <0.001 | -0.64 (0.68) | 0.34 |
| Grip strength, women, kg | | | | | | |  |
| Baseline | 297 | 22.40 (0.37) |  | 21.59 (0.36) |  |  |  |
| Year 2 | 263 | 15.69 (0.34) |  | 15.35 (0.33) |  |  |  |
| 2-year change |  | -6.71 (0.32) | <0.001 | -6.24 (0.31) | <0.001 | -0.47 (0.45) | 0.29 |
| SPPB score | | | | | | |  |
| Baseline | 569 | 11.20 (0.06) |  | 11.04 (0.06) |  |  |  |
| Year 2 | 509 | 11.01 (0.07) |  | 10.92 (0.07) |  |  |  |
| 2-year change |  | -0.19 (0.07) | 0.004 | -0.12 (0.06) | 0.06 | -0.07 (0.09) | 0.43 |
| Normal walking speed, m/s | | | | | | |  |
| Baseline | 599 | 1.20 (0.01) |  | 1.20 (0.01) |  |  |  |
| Year 2 | 529 | 1.16 (0.01) |  | 1.16 (0.01) |  |  |  |
| 2-year change |  | -0.04 (0.01) | <0.001 | -0.04 (0.01) | <0.001 | 0.00 (0.01) | 0.78 |
| Fast walking speed, m/s | | | | | | |  |
| Baseline | 587 | 1.68 (0.01) |  | 1.66 (0.01) |  |  |  |
| Year 2 | 522 | 1.65 (0.02) |  | 1.63 (0.01) |  |  |  |
| 2-year change |  | -0.02 (0.01) | 0.04 | -0.02 (0.01) | 0.01 | 0.00 (0.02) | 0.80 |
| Standing balance, s | | | | | | |  |
| Baseline | 595 | 29.05 (1.7) |  | 29.01 (0.16) |  |  |  |
| Year 2 | 526 | 29.33 (0.14) |  | 29.43 (0.13) |  |  |  |
| 2-year change |  | 0.28 (0.17) | 0.09 | 0.41 (0.16) | 0.01 | -0.13 (0.24) | 0.58 |
| Chair stands, s | | | | | | |  |
| Baseline | 573 | 11.11 (0.15) |  | 11.70 (0.14) |  |  |  |
| Year 2 | 513 | 11.89 (0.21) |  | 12.05 (0.20) |  |  |  |
| 2-year change |  | 0.78 (0.18) | <0.001 | 0.34 (0.18) | 0.05 | 0.44 (0.25) | 0.08 |
| Timed Up and Go, s | | | | | | |  |
| Baseline | 597 | 8.33 (0.08) |  | 8.33 (0.08) |  |  |  |
| Year 2 | 522 | 7.91 (0.09) |  | 7.89 (0.09) |  |  |  |
| 2-year change |  | -0.42 (0.08) | <0.001 | -0.44 (0.08) | <0.001 | 0.01 (0.11) | 0.91 |

Abbreviations: SPPB, Short Physical Performance Battery

Supplementary Table 3. Two-year changes in Physical Performance Measures, according to randomized treatment assignment, adjusted for age, sex, and treatment group

|  | N | Cocoa Extract | | Cocoa Extract Placebo | | Treatment effect | | Multivitamin/ Multimineral | | Multivitamin/ Multimineral Placebo | | Treatment effect | |
| --- | --- | --- | --- | --- | --- | --- | --- | --- | --- | --- | --- | --- | --- |
|  |  | Mean (SE) | p-value | Mean (SE) | p-value | Mean (SE) | p-value | Mean (SE) | p-value | Mean (SE) | p-value | Mean (SE) | p-value |
| Grip Strength/Body Mass Index, kg/kg/m^2^ | | | | | | | | | | | | | |
| Baseline | 603 | 1.09 (0.02) |  | 1.10 (0.02) |  |  |  | 1.11 (0.02) |  | 1.08 (0.02) |  |  |  |
| Year 2 | 535 | 0.75 (0.02) |  | 0.78 (0.02) |  |  |  | 0.77 (0.02) |  | 0.76 (0.02) |  |  |  |
| 2-year change |  | -0.34 (0.01) | <0.001 | -0.32  (0.01) | <0.001 | -0.02 (0.02) | 0.35 | -0.34 (0.01) | <0.001 | -0.32 (0.01) | <0.001 | -0.02 (0.02) | 0.17 |
| Grip Strength/Body Mass Index, men, kg/kg/m^2^ | | | | | | | | | | | | | |
| Baseline | 306 | 1.35 (0.03) |  | 1.35 (0.03) |  |  |  | 1.35 (0.03) |  | 1.35 (0.03) |  |  |  |
| Year 2 | 272 | 0.92 (0.02) |  | 0.93 (0.03) |  |  |  | 0.92 (0.03) |  | 0.94 (0.02) |  |  |  |
| 2-year change |  | -0.42 (0.02) | <0.001 | -0.42 (0.02) | <0.001 | -0.01 (0.03) | 0.81 | -0.43 (0.02) | <0.001 | -0.41 (0.02) | <0.001 | -0.02 (0.03) | 0.42 |
| Grip Strength/Body Mass Index, women, kg/kg/m^2^ | | | | | | | | | | | | | |
| Baseline | 297 | 0.83 (0.02) |  | 0.85 (0.02) |  |  |  | 0.87 (0.02) |  | 0.81 (0.02) |  |  |  |
| Year 2 | 263 | 0.58 (0.02) |  | 0.62 (0.02) |  |  |  | 0.62 (0.02) |  | 0.58 (0.02) |  |  |  |
| 2-year change |  | -0.24 (0.01) | <0.001 | -0.23 (0.01) | <0.001 | -0.01 (0.02) | 0.56 | -0.25 (0.01) | <0.001 | -0.23 (0.01) | <0.001 | -0.03 (0.02) | 0.16 |
| Semi-Tandem Stand, s | | | | | | | | | | | | | |
| Baseline | 590 | 10.00(0.02) |  | 9.98 (0.02) |  |  |  | 10.00 (0.02) |  | 9.98 (0.02) |  |  |  |
| Year 2 | 525 | 9.98 (0.01) |  | 10.00 (0.01) |  |  |  | 10.00 (0.01) |  | 9.99 (0.01) |  |  |  |
| 2-year change |  | -0.01 (0.02) | 0.52 | 0.03 (0.02) | 0.22 | -0.04 (0.03) | 0.19 | 0.002 (0.02) | 0.91 | 0.01 (0.02) | 0.63 | -0.01 (0.03) | 0.80 |
| Tandem Stand, s | | | | | | | | | | | | | |
| Baseline | 579 | 9.25 (0.10) |  | 9.51 (0.10) |  |  |  | 9.47 (0.11) |  | 9.31 (0.10) |  |  |  |
| Year 2 | 511 | 9.78 (0.08) |  | 9.68 (0.07) |  |  |  | 9.71 (0.08) |  | 9.74 (0.07) |  |  |  |
| 2-year change |  | 0.52 (0.12) | <0.001 | 0.17 (0.11) | 0.14 | 0.35 (0.16) | 0.03 | 0.25 (0.12) | 0.04 | 0.43 (0.11) | <0.001 | -0.19 (0.16) | 0.25 |

Supplementary Table 4. Effect of Multivitamin/Multimineral Supplementation vs. Placebo on Physical Performance Measures in Subgroups

|  | Multivitamin/Multimineral | | | Multivitamin/Multimineral Placebo | | | P-value, treatment effect | P for interaction |
| --- | --- | --- | --- | --- | --- | --- | --- | --- |
| Subgroup | N | 2-year change (SE) | p-value | N | 2-year change (SE) | p-value |  |  |
| *Grip Strength, kg* | | | | | | | | |
| Sex |  |  |  |  |  |  |  | 0.83 |
| Men | 147 | -12.23 (0.49) | <0.001 | 159 | -11.58 (0.47) | <0.001 | 0.34 |  |
| Women | 142 | -6.71 (0.32) | <0.001 | 155 | -6.23 (0.31) | <0.001 | 0.29 |  |
| Age |  |  |  |  |  |  |  | 0.82 |
| <median (69.2 years) | 151 | -10.57 (0.49) | <0.001 | 150 | -9.94 (0.48) | <0.001 | 0.36 |  |
| ≥median | 138 | -8.44 (0.46) | <0.001 | 164 | -8.88 (0.33) | <0.001 | 0.49 |  |
| Body Mass Index |  |  |  |  |  |  |  | 0.32 |
| <median (26.5 kg/m^2^) | 145 | -8.69 (0.46) | <0.001 | 156 | -8.56 (0.45) | <0.001 | 0.83 |  |
| ≥median | 144 | -10.41 (0.49) | <0.001 | 158 | -9.33 (0.46) | <0.001 | 0.11 |  |
| Leisure-time physical activity and stair climbing, total MET |  |  |  |  |  |  |  | 0.71 |
| <median (20.0 hours/week) | 139 | -8.82 (0.47) | <0.001 | 160 | -8.14 (0.46) | <0.001 | 0.29 |  |
| ≥median | 149 | -10.14 (0.48) | <0.001 | 151 | -9.81 (0.48) | <0.001 | 0.64 |  |
| History of ≥1 fall in the last year |  |  |  |  |  |  |  | 0.08 |
| Yes | 86 | -8.24 (0.59) | <0.001 | 91 | -8.90 (0.57) | <0.001 | 0.43 |  |
| No | 201 | -10.11 (0.41) | <0.001 | 222 | -8.95 (0.39) | <0.001 | 0.04 |  |
| History of ≥2 falls in the last year |  |  |  |  |  |  |  | 0.87 |
| Yes | 35 | -10.54 (1.02) | <0.001 | 37 | -9.72 (0.97) | <0.001 | 0.56 |  |
| No | 252 | -9.44 (0.36) | <0.001 | 276 | -8.84 (0.35) | <0.001 | 0.23 |  |
| General Health, self-reported |  |  |  |  |  |  |  | 0.20 |
| Excellent | 106 | -9.64 (0.56) | <0.001 | 105 | -9.01 (0.56) | <0.001 | 0.42 |  |
| Very Good | 135 | -9.42 (0.49) | <0.001 | 159 | -9.38 (0.46) | <0.001 | 0.95 |  |
| Good | 39 | -10.03 (0.93) | <0.001 | 40 | -6.96 (0.87) | <0.001 | 0.02 |  |
| Fair | 4 | -10.31 (3.18) | 0.03 | 5 | -7.35 (3.17) | 0.081 | 0.55 |  |
| Prior Multivitamin Use |  |  |  |  |  |  |  | 0.46 |
| Yes | 114 | -9.24 (0.51) | <0.001 | 177 | -9.06 (0.51) | <0.001 | 0.81 |  |
| No | 175 | -9.73 (0.45) | <0.001 | 196 | -8.85 (0.42) | <0.001 | 0.15 |  |
| Randomized to Cocoa Extract |  |  |  |  |  |  |  | 0.76 |
| Yes | 154 | -9.72 (0.46) | <0.001 | 145 | -9.31 (0.47) | <0.001 | 0.53 |  |
| No | 135 | -9.33 (0.49) | <0.001 | 169 | -8.64 (0.45) | <0.001 | 0.30 |  |
| *SPPB score* | | | | | | | | |
| Sex |  |  |  |  |  |  |  | 0.78 |
| Men | 147 | -0.31 (0.09) | <0.001 | 159 | -0.21 (0.08) | 0.01 | 0.44 |  |
| Women | 142 | -0.06(0.10) | 0.53 | 155 | -0.02 (0.10) | 0.86 | 0.74 |  |
| Age |  |  |  |  |  |  |  | 0.71 |
| <median (69.2 years) | 151 | -0.17 (0.09) | 0.05 | 150 | -0.13 (0.08) | 0.12 | 0.74 |  |
| ≥median | 138 | -0.21 (0.10) | 0.04 | 164 | -0.10 (0.10) | 0.29 | 0.43 |  |
| Body Mass Index |  |  |  |  |  |  |  | 0.67 |
| <median (26.5 kg/m^2^) | 145 | -0.26 (0.09) | 0.003 | 156 | -0.14 (0.09) | 0.09 | 0.36 |  |
| ≥median | 144 | -0.12 (0.10) | 0.24 | 158 | -0.09 (0.09) | 0.35 | 0.82 |  |
| Leisure-time physical activity and stair climbing, total MET |  |  |  |  |  |  |  | 0.30 |
| <median (20.0 hours/week) | 139 | -0.16 (0.11) | 0.14 | 160 | -0.19 (0.10) | 0.06 | 0.83 |  |
| ≥median | 149 | -0.22 (0.08) | 0.01 | 151 | -0.07 (0.08) | 0.42 | 0.18 |  |
| History of ≥1 fall in the last year |  |  |  |  |  |  |  | 0.98 |
| Yes | 86 | -0.30 (0.13) | 0.02 | 91 | -0.22 (0.12) | 0.07 | 0.67 |  |
| No | 201 | -0.15 (0.08) | 0.05 | 222 | -0.07 (0.07) | 0.35 | 0.46 |  |
| History of ≥2 falls in the last year |  |  |  |  |  |  |  | 0.49 |
| Yes | 35 | -0.49 (0.21) | 0.03 | 37 | -0.24 (0.20) | 0.25 | 0.39 |  |
| No | 252 | -0.16 (0.07) | 0.03 | 276 | -0.10 (0.07) | 0.14 | 0.56 |  |
| General Health, self-reported |  |  |  |  |  |  |  | 0.48 |
| Excellent | 106 | -0.21 (0.10) | 0.03 | 105 | -0.25 (0.10) | 0.01 | 0.74 |  |
| Very Good | 135 | -0.21 (0.10) | 0.04 | 159 | -0.01 (0.09) | 0.94 | 0.15 |  |
| Good | 39 | 0.03 (0.22) | 0.89 | 40 | -0.10 (0.21) | 0.62 | 0.67 |  |
| Fair | 4 | *** | *** | 5 | *** | *** |  |  |
| Prior Multivitamin Use |  |  |  |  |  |  |  | 0.79 |
| Yes | 114 | -0.13 (0.10) | 0.22 | 117 | -0.09 (0.10) | 0.39 | 0.79 |  |
| No | 175 | -0.23 (0.09) | 0.009 | 196 | -0.14 (0.08) | 0.09 | 0.47 |  |
| Randomized to Cocoa Extract |  |  |  |  |  |  |  | 0.10 |
| Yes | 154 | -0.05 (0.10) | 0.59 | 145 | -0.12 (0.10) | 0.21 | 0.60 |  |
| No | 135 | -0.35 (0.09) | <0.001 | 169 | -0.12 (0.08) | 0.16 | 0.06 |  |
| *Normal Walking Speed, m/s* | | | | | | | | |
| Sex |  |  |  |  |  |  |  | 0.49 |
| Men | 147 | -0.01 (0.01) | 0.56 | 159 | -0.02 (0.01) | 0.12 | 0.50 |  |
| Women | 142 | -0.07 (0.01) | <0.001 | 155 | -0.06 (0.01) | <0.001 | 0.77 |  |
| Age |  |  |  |  |  |  |  | 0.83 |
| <median (69.2 years) | 151 | -0.03 (0.01) | 0.04 | 150 | -0.03 (0.01) | 0.03 | 0.98 |  |
| ≥median | 138 | -0.05 (0.01) | <0.001 | 164 | -0.05 (0.01) | <0.001 | 0.73 |  |
| Body Mass Index |  |  |  |  |  |  |  | 0.35 |
| <median (26.5 kg/m^2^) | 145 | -0.05 (0.01) | <0.001 | 156 | -0.04 (0.01) | 0.001 | 0.62 |  |
| ≥median | 144 | -0.03 (0.01) | 0.03 | 158 | -0.04 (0.01) | <0.001 | 0.40 |  |
| Leisure-time physical activity and stair climbing, total MET |  |  |  |  |  |  |  | 0.24 |
| <median (20.0 hours/week) | 139 | -0.03 (0.01) | 0.02 | 160 | -0.05 (0.01) | <0.001 | 0.37 |  |
| ≥median | 149 | -0.04 (0.01) | <0.001 | 151 | -0.03 (0.01) | 0.004 | 0.44 |  |
| History of ≥1 fall in the last year |  |  |  |  |  |  |  | 0.19 |
| Yes | 86 | -0.04 (0.02) | 0.01 | 91 | -0.02 (0.02) | 0.18 | 0.36 |  |
| No | 201 | -0.04 (0.01) | <0.001 | 222 | -0.05 (0.01) | <0.001 | 0.36 |  |
| History of ≥2 falls in the last year |  |  |  |  |  |  |  | 0.34 |
| Yes | 35 | -0.05 (0.03) | 0.05 | 37 | -0.02 (0.03) | 0.40 | 0.41 |  |
| No | 252 | -0.04 (0.01) | <0.001 | 276 | -0.04 (0.01) | <0.001 | 0.61 |  |
| General Health, self-reported |  |  |  |  |  |  |  | 0.68 |
| Excellent | 106 | -0.04 (0.01) | 0.01 | 105 | -0.04 (0.01) | 0.002 | 0.76 |  |
| Very Good | 135 | -0.04 (0.01) | 0.002 | 159 | -0.04 (0.01) | <0.001 | 0.78 |  |
| Good | 39 | -0.03 (0.03) | 0.25 | 40 | -0.02 (0.03) | 0.46 | 0.75 |  |
| Fair | 4 | -0.11 (0.04) | 0.09 | 5 | -0.26 (0.06) | 0.02 | 0.12 |  |
| Prior Multivitamin Use |  |  |  |  |  |  |  | 0.16 |
| Yes | 114 | -0.05 (0.01) | <0.001 | 117 | -0.03 (0.01) | 0.01 | 0.35 |  |
| No | 175 | -0.03 (0.01) | 0.01 | 196 | -0.04 (0.01) | <0.001 | 0.27 |  |
| Randomized to Cocoa Extract |  |  |  |  |  |  |  | 0.45 |
| Yes | 154 | -0.03 (0.01) | 0.03 | 145 | -0.04 (0.01) | 0.002 | 0.50 |  |
| No | 135 | -0.05 (0.01) | <0.001 | 169 | -0.04 (0.01) | <0.001 | 0.71 |  |

Abbreviations: MET, metabolic equivalent of task; SPPB, Short Physical Performance Battery
